# Photophysical implications of ring fusion, linker length, and twisting angle in a series of perylenediimide–thienoacene dimers†
†Electronic supplementary information (ESI) available. See DOI: 10.1039/d0sc02862b


**DOI:** 10.1039/d0sc02862b

**Published:** 2020-07-01

**Authors:** Ariel A. Leonard, Martín A. Mosquera, Leighton O. Jones, Zhengxu Cai, Thomas J. Fauvell, Matthew S. Kirschner, David J. Gosztola, George C. Schatz, Richard D. Schaller, Luping Yu, Lin X. Chen

**Affiliations:** a Department of Chemistry , Northwestern University , 2145 Sheridan Road , Evanston , Illinois 60208 , USA . Email: l-chen@northwestern.edu; b Beijing Key Laboratory of Construction Tailorable Advanced Functional Materials and Green Applications , School of Materials Science & Engineering , Beijing Institute of Technology , Beijing 100081 , China; c Department of Chemistry and James Frank Institute , The University of Chicago , 929 East 57th Street , Chicago , Illinois 60637 , USA; d Center for Nanoscale Materials , Argonne National Laboratory , 9700 South Cass Avenue Lemont , Illinois 60439 , USA; e Chemical Sciences and Engineering Division , Argonne National Laboratory , 9700 South Cass Avenue, Lemont , Illinois 60439 , USA . Email: lchen@anl.gov

## Abstract

Perylenediimide (PDI) derivatives have been widely studied as electron acceptor alternatives to fullerenes in organic photovoltaics (OPVs) because of their tunable absorption in the visible range, inexpensive synthesis, and photochemical stability. A common motif for improving device efficiency involves joining multiple PDIs together through electron-rich linkers to form a twisted acceptor–donor–acceptor molecule. Molecular features such as ring fusion are further employed to modify the structure locally and in films. These synthetic efforts have greatly enhanced OPV device efficiencies, however it remains unclear how the increasingly elaborate structural modifications affect the photophysical processes integral to efficient photon-to-charge conversion. Here we carry out a systematic study of a series of PDI dimers with thienoacene linkers in which the twist angle, linker length, and degree of ring fusion are varied to investigate the effects of these structural features on the molecular excited states and exciton recombination dynamics. Spectroscopic characterization of the dimers suggest that ring fusion causes greater coupling between the donor and acceptor components and greatly enhances the lifetime of a thienoacene to PDI charge transfer state. The lifetime of this CT state also correlates well with the linker–PDI dihedral angle, with smaller dihedral angle resulting in longer lifetime. DFT and two-photon absorption TDDFT calculations were developed in-house to model the ground state and excited transitions, providing theoretical insight into the reasons for the observed photophysical properties and identifying the charge transfer state in the excited state absorption spectra. These results highlight how the longevity of the excited state species, important for the efficient conversion of excitons to free carriers in OPV devices, can be chemically tuned by controlling ring fusion and by using steric effects to control the relative orientations of the molecular fragments. The results provide a successful rationalization of the behavior of solar cells involving these acceptor molecules.

## Abstract 2

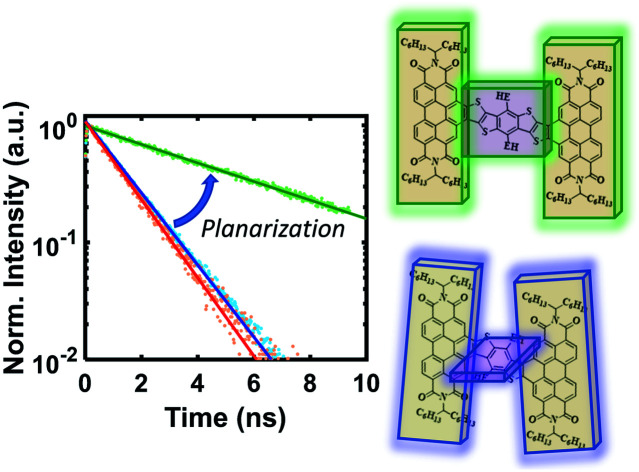
Ring fusion and conjugated bridge length dependent exciton dynamics and electronic coupling in a series of perylenediimide dimers with acceptor–donor–acceptor arrangement are investigated by ultrafast optical spectroscopy and TDDFT calculations.

## Introduction

The development of organic photovoltaic (OPV) electron acceptors has progressed rapidly in the last several years, with power conversion efficiencies (PCEs) exceeding 18% in conjugated polymer and small molecule OPV devices.^1^–^4^ Most OPV devices utilize a photoactive layer of intermixed electron donating and accepting materials,^5^ known as a bulk heterojunction (BHJ), to generate large areas of the donor–acceptor interface where charge separation occurs. Much of the recent OPV improvement has come from the electron acceptor materials, namely non-fullerene acceptors.^6^ Historically, fullerene derivatives have been the most commonly used and extensively studied electron accepting materials,^7^–^9^ however high cost, photochemical instability, and challenges with processability have led the field to pivot towards non-fullerene alternatives.^10^,^11^ Among these alternatives, perylenediimide (PDI) derivatives (particularly those connecting multiple PDI blocks), are appealing candidates because they are comparatively inexpensive to produce, photochemically stable, and synthetically tunable for optimal HOMO/LUMO overlap with the electron donor.^12^,^13^

**Fig. 1:**
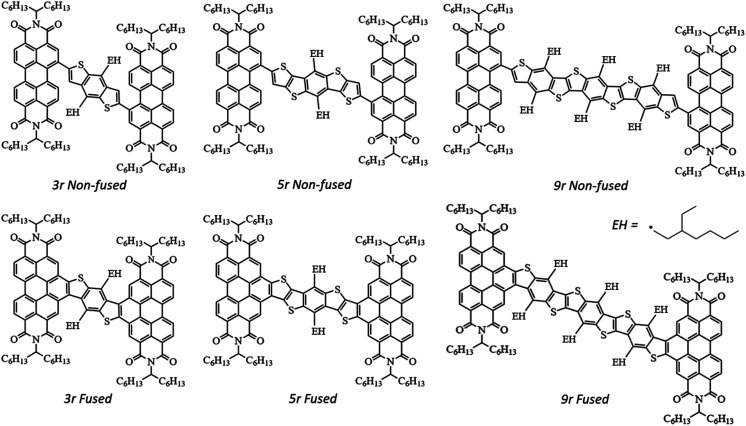
PDI dimers with thienoacene linkers of variable length. The connectivity between the thienoacene and the PDIs is single bond (top) and fused-ring aromatic (bottom). The side chain on the thienoacene linker is an ethylhexane group, denoted EH.

The highest performing PDI acceptors share several key molecular features. First, multiple PDI molecules are often linked together to form twisted dimers, trimers, and tetramers.^6^,^13^–^25^ The twisted structure inhibits π–π interactions between neighboring PDIs which are otherwise prone to aggregate into undesirable micrometer-scale crystalline domains, reducing the donor–acceptor interfacial area.^26^–^28^ It also accommodates PDI interface with electron donors in different orientations, somewhat mimicking the isotropic interactions credited as key to efficient donor–acceptor charge transfer in fullerene based active layers.^28^,^29^ Second, taking cues from the electron push–pull structures in many recent conjugated polymers which have proven to be effective electron donors in OPV active layers and OLEDs,^30^,^31^ the PDI units are joined together with an electron donating linker such as a phenol, thiophene, or benzodithiophene group.^6^,^15^–^18^,^23^,^32^–^34^ PDI and the electron donating bridging blocks combine to form an acceptor–donor–acceptor (A–D–A) complex, which promotes intramolecular charge transfer (Fig. 1). Third, ring fusion, by which the bridging linker and the PDI are connected *via* a phenyl group, is employed to fix the relative orientations of the PDIs and promote exciton delocalization across the core of the molecule. Ring-fused complexes have been shown to exhibit superior efficiency to their non-fused counterparts when tested in OPV devices.^15^–^18^,^34^,^35^

Great strides have been made in improving device efficiency through such synthetic routes, however there have been few studies^36^–^39^ focused on electronic processes in these increasingly complex molecules. Photoexcitation is the first in a series of ultrafast processes that lead to exciton generation, charge separation, and charge carrier transport in photovoltaic materials, so understanding the molecular excited state/excitonic properties can be crucial for controlling photophysical processes and informing molecular design.^39^–^44^ Electronic coupling between the PDI moieties and the linker, which can conjugate with the PDI moieties to different extents, lead to emerging properties in PDI dimers and oligomers. For example, the lowest energy transition that dominates many of these A–D–A conjugated organic molecules has strong charge transfer (CT) character with partial positive and negative charges delocalized across different moieties.^45^,^46^

In an effort to differentiate between the contributions from various molecular features – ring fusion, linker length, and twisting angle between PDI moieties – we perform a systematic study on the excited state properties of a series of PDI dimers shown in Fig. 1.^15^,^33^ These dimers have conjugated thienoacene linkers^47^,^48^ composed of 3, 5, and 9 aromatic rings (“**3r**”, “**5r**”, and “**9r**”), with either single bond (“non-fused”) or aromatic (“fused”) connectivity between the linker and the PDI. Also, there is an ethylhexane group (denoted EH in Fig. 1) that sterically interacts with the PDI moieties in **3r** and **9r** but not **5r**, resulting in structural effects that will prove important. We use ultrafast spectroscopic techniques and computational modeling with Density Functional Theory (DFT) to investigate how these changes in molecular structure affect the electronic structure, excited state formation, and dynamics. Using this series, we can identify trends associated with ring fusion, linker length, and twist angle to further fundamental understanding regarding the rational design of this family of electron acceptors.

### Steady state characterization

**Fig. 2:**
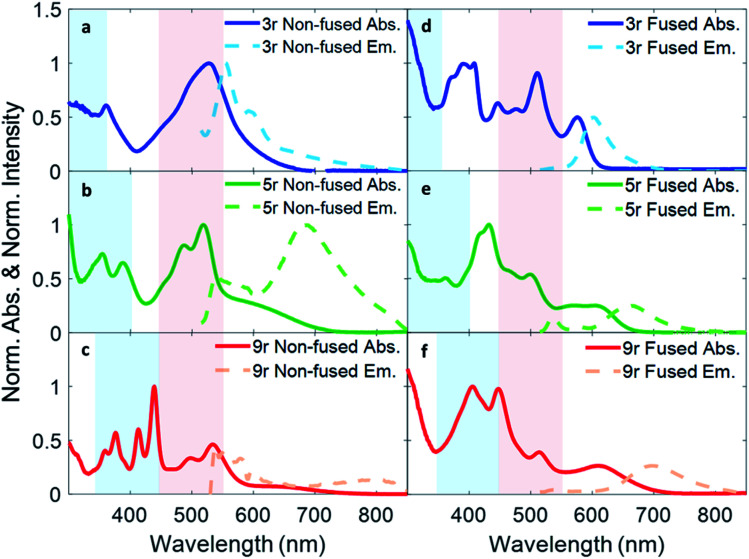
Optical absorption and emission spectra of (a) non-fused **3r**, (b) non-fused **5r**, (c) non-fused **9r**, (d) fused **3r**, (e) fused **5r**, and (f) fused **9r** dimers. The light blue color shading indicates the absorption range of the three thienoacene components used in the synthesis, while the red shading indicates absorption of PDI monomer in solution.

Absorption and fluorescence spectra for all six dimers in chlorobenzene are shown in Fig. 2. Numerous absorption peaks arise from the vibronic transitions of the thienoacene and PDI components. The PDI monomer absorbs in the range of 450–550 nm,^49^,^50^ while the **3r**, **5r**, and **9r** thienoacenes absorb in the ranges of 250–350 nm, 300–400 nm, and 350–450 nm, respectively.^51^ These absorption ranges are indicated with blue and red shadings in Fig. 2 for reference. The apparent contributions of these transitions vary greatly in the non-fused *versus* the fused dimers.

In the non-fused series (Fig. 2a–c), the regions of high optical density for the dimers correspond well with the PDI and thienoacene component absorptions, indicative of weak coupling between the linker and the PDI (limited by rotation of the single bond connecting them), and between the PDIs. In the **3r** and **5r** spectra the PDI and thienoacene features appear broadened, possibly due to the collective contributions of multiple low energy molecular conformations that become more evident in later time-resolved measurements. The non-fused **9r** dimer exhibits the least electronic aberration from the monomer components, retaining the vibronic structure of both the thienoacene and PDI absorptions.^49^,^51^ New in all three non-fused spectra is a broad tail of absorption to lower energy extending past 600 nm that cannot be accounted for by either the thienoacene or PDI absorptions. This feature likely results from an intramolecular CT transition, based on additional UV-Vis measurements in chloroform showing a solvatochromic shift for this transition (ESI Fig. 1†). The slightly more polar chloroform provides solvent stabilization of the CT dipole, resulting in an absorption shift^52^ to lower energy. The thienoacene and PDI fragments retain some independent character in the non-fused dimers, however there is still modest coupling between them that allows for delocalization of the electron density across the linker–PDI bond.

In the fused dimer series (Fig. 2d–f), the ring fusion leads to enhanced electronic coupling between the thienoacene linker and PDI moieties, as evidenced by drastic changes in the absorption spectral features from those of their non-fused counterparts. The origins of these new peaks and peaks shifts are not immediately clear as their positions and shapes do not bear apparent correspondence to those of the non-fused dimers or the building blocks. Additionally, the lowest energy transition CT peak – and the solvatochromic shift – is more prominent in the fused dimers as compared to the non-fused dimers, suggesting enhanced conjugation between the linker and PDI moieties *via* the aromatic bridge. In all six dimers, the charge transfer peak progressively decreases in energy with lengthening linker because of an increase in the linker HOMO energy level associated with the increased conjugation length, while the PDI LUMO energy level stays comparatively constant.^47^

In order to quantify the energetic evolution of molecular orbitals (MOs) as well as the electronic coupling between the bridge and the PDI moieties as a function of the linker length, we performed linear-response time-dependent DFT calculations to estimate the ground-state and excited state absorption spectra of the **3r** and **5r** PDI dimers. For the **9r** systems we computed the ground-state orbitals and a few excited state roots to analyse the low-lying transitions.

**Fig. 3:**
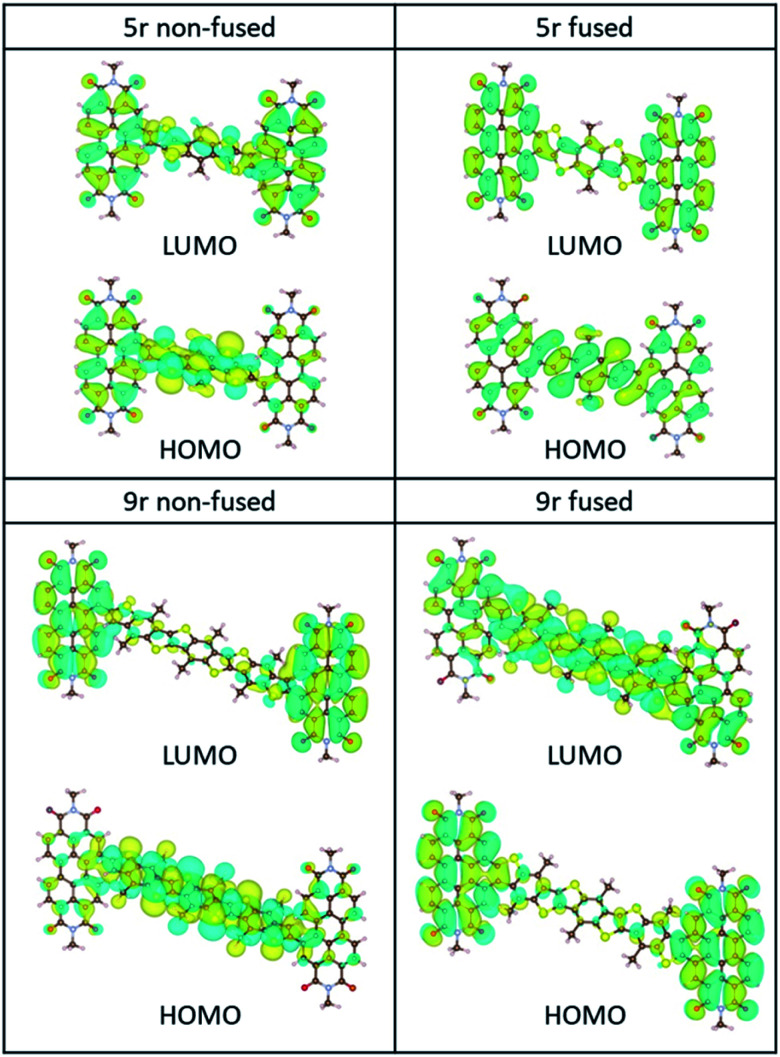
HOMO/LUMO orbitals of the non-fused and fused **5r** and **9r** systems, computed at the B3LYP/6-31G* level.

The ground-state HOMO–LUMO pairs are displayed in Fig. 3. As expected, the lowest energy transition in these conjugated aromatic systems are dominated by the HOMO–LUMO transitions. These transitions exhibit CT character, as depicted by electron density shift from the bridge to the PDI ends. Interestingly, the fused **9r** molecule is an exception, and the CT direction is reversed with respect to the other systems. This reversal is attributed to the increased length of the linker, which lowers the LUMO energy level of the linker to within close proximity to that of the PDI, facilitating greater mixing between the two. A similar trend was observed in our previous publication on push–pull conjugated polymers, in which lengthening the donor block fully flipped the relative positions of the donor and acceptor LUMOs, and in fact localized the exciton on the donor block.^53^

The incongruent behaviour between the non-fused and fused **9r** dimers can be explained by examining the conjugation along the entire length of the aromatic system. In the non-fused system, although the lengthening of the bridge lowers the LUMO energy level with respect to the **3r** and **5r** dimers, its conjugation length is limited to the 9-ring segment. Strong conjugation with the PDI moieties is limited by the severely non-planar relative aromatic plane orientation between the **9r** bridge and the PDI ends. By contrast, in the fused **9r** system, the additional phenyl group on either end of the bridge and the more planar conformation of the entire molecule extends the conjugation length significantly, leading to a strong coupling between PDI units and linker. Additional molecular orbital plots and assignments of specific ground state transitions are shown in ESI Fig. 2 and 3.†

Our calculated ground-state absorption spectra (ESI Fig. 2 and 3†) for the **3r** and **5r** fused molecules show qualitative agreement with experiment in the PDI and linker regions. The computed spectra of the **3r** and **5r** non-fused systems, however, show that the CT absorption energy is underestimated. Furthermore, although the calculations show absorption peaks in the monomer PDI and linker regions, the relative heights do not match the experimental spectra. This artificial behavior is caused by the choice of exchange-correlation functional approximation and the lack of computed vibronic information about the system, which is out of scope, but could explain the deviations. We chose the functional B3LYP as it gives a balanced description over the experimental window of wavelengths. Additionally, discrepancies may originate from the differences in equilibrium bridge–PDI dihedral angle *in vacuo* (as modeled for the calculations) *versus* in solution (as for the experiments). Since the fused bridge has little freedom to rotate, the structures *in vacuo* and in solution will be more similar in the fused dimers than in the non-fused ones, and indeed the fused structures have better agreement between calculation and experiment.

Despite these observations, the calculations agree with the experimental trend that the CT transition of the **9r** non-fused molecule red-shifts more with respect to the **5r** non-fused than its peer, the **9r** fused system (ESI Table 1†), and is consistent with the other shifts observed experimentally (between the **3r** and **5r** non-fused molecules and between the **3r** and **5r** fused ones). In addition, the molecular orbitals shown in ESI Fig. 2 and 3† for the fused dimers appear to be more delocalized than for the non-fused dimers, particularly for the linker and PDI transitions at ∼350 nm and ∼500 nm, respectively.

Fluorescence spectra are plotted in dotted lines on the same axes as the absorption spectra in Fig. 1. An excitation wavelength of 500 nm was used to selectively target the PDI absorption, since 500 nm is too low in energy to be absorbed by the thienoacene linkers. Emissions from the PDI singlet and CT states are present to various extents in each of the spectra. The CT emission corresponds well to HOMO–LUMO energy gaps previously measured *via* cyclic voltammetry.^15^ The **3r** and **5r** non-fused dimers emit from both the PDI singlet state (560–575 nm) and the CT state (broadtail on the red side of the spectrum), implying that in some conformations the exciton remains localized on the PDI and other conformations undergo charge transfer. In contrast, the fused dimers predominantly emit from the CT state, implying that in most cases the exciton relaxes to the lowest energy CT band before returning to the ground state, likely aided by increased coupling between the PDI and thienoacene moieties.

### Characterization of charge transfer lifetime with time-resolved emission

**Fig. 4:**
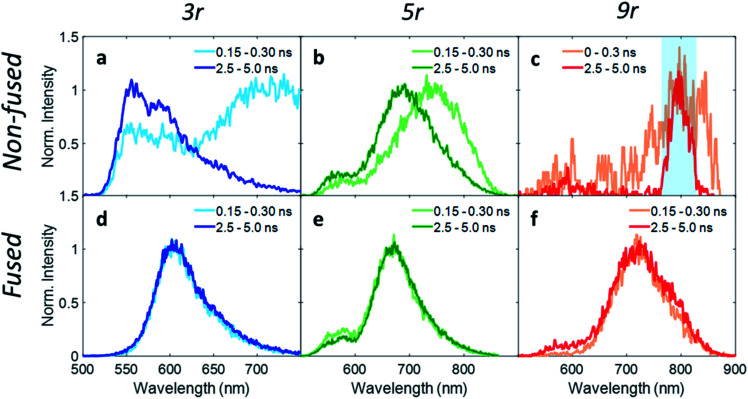
Wavelength traces from streak camera measurements with 500 nm pump at early (0.3 ns) and late (4.5 ns) times for (a) non-fused **3r**, (b) non-fused **5r**, (c) non-fused **9r**, (d) fused **3r**, (e) fused **5r**, (f) fused **9r** dimers. In the non-fused **9r** dimer, the charge transfer emission is very weak due to strong fluorescence quenching, and the 800 nm fundamental beam (highlighted in blue) is visible.

In order to investigate exciton dynamics in the PDI dimers, their time-resolved emission spectra were measured with streak camera detection, which simultaneously provides high spectral and temporal resolution. The sample was excited at 500 nm to selectively excite the PDI moiety. Spectra averaged over early and late time windows (0.15–0.30 and 2.5–5.0 ns after excitation) are plotted in Fig. 4, which highlight the evolution of the excited state species.

For the **3r** non-fused dimer, there are two apparent transitions: a shorter-lived emission between 650 and 750 nm from the CT state, and a longer-lived emission between 550 and 650 nm from the PDI singlet state. The assignments are based on steady-state emission of the dimers and PDI monomer, as discussed above. We find that each of these features decays at a different rate, which becomes evident upon examination of the early and late time traces shown in Fig. 4a, and the change in relative prominence of the two features thereof. Kinetics were extracted by integrating the full spectrum and fitting the decay, which exhibited clear biexponential behavior with decay constants of 0.34 ns and 3.47 ns corresponding to the CT and PDI singlet states, respectively. In contrast, the **3r** fused dimer has a single emission peak consistent with the CT emission observed in the steady state fluorescence spectrum. The entire emission peak evolves uniformly in time and decays monoexponentially with a time constant of 1.41 ns, suggesting formation of a single excited state species.

We suggest that the dual emission observed in the non-fused dimer arises from conformational heterogeneity, with some conformations favoring delocalization of the exciton to the CT state and others favoring localization of the exciton on the PDI. The lifetime for the PDI singlet emission observed here is consistent with known values,^12^,^49^ and while CT lifetimes can be quite variable depending on the specific composition, related compounds have shown CT occurring over timescales of similar magnitude.^36^ There is also precedence for this type of dual emissive behavior in twisted A–D–A complexes.^37^,^54^

Correspondingly, the uniform emission observed in the fused dimer is likely a consequence of the locked structure imparted by the ring fusion. It is also of note that the fused CT transition has a lifetime of more than a factor of three greater than that of the non-fused CT, despite the two being very similar in energy (2.06 eV *vs.* 1.95 eV).^15^ This could be because the molecular orbitals in the fused dimer are more delocalized, stabilizing the exciton; in addition the comparatively flexible structure of the non-fused dimer allows for more non-radiative internal conversion pathways that can accelerate the rate of exciton relaxation.

Extending these observations to the rest of the dimer series, it is apparent that like the **3r** non-fused dimer, the **5r** non-fused dimer has multiple emissive states: two main emissions at 690 and 750 nm, and a third weaker one near 550 nm. Strong fluorescence quenching in the **9r** non-fused dimer precludes in-depth analysis, however there is some fluorescence intensity visible at early time in the ranges of 500–600 nm and 750–850 nm, likely corresponding to the PDI singlet and CT emissions in the same manner as discussed for the **3r** non-fused dimer. The sharp peak seen at 800 nm is scatter of the 800 nm fundamental beam. In the fused **5r** and **9r** spectra, there is a small amount of PDI emission in the 500–600 nm range; however, the main emission occurs in the 600–900 nm range from the CT state. In the fused **9r** spectrum there appears to be an additional fast decay on the blue side of the spectrum (not fitted due to weak emission) which could represent evolution from PDI singlet to CT states. The lifetimes of the various states are given in Table 1.

**Table 1:** Dimer lifetimes. Lifetimes of the dimer excited states, as measured by fsTA, streak camera, and nsTA. Global analysis was used for the fsTA analysis. Lifetimes from streak camera measurements were determined using an exponential fitting algorithm

Sample	fsTA				Streak camera		nsTA	
	*τ*_1_ (ps)	*τ*_2_ (ps)	*τ*_3_ (ps)	*τ*_4_ (ps)	*τ*_CT_ (ps)	*τ*_PDI_ (ps)	*τ*_3_ (ps)	*τ*_4_ (μs)
**3r** non-fused	13.1	—	386	Long time	341 ± 7	3466 ± 81	—	11.7 ± 0.2
**5r** non-fused	9.9	64	727	Long time	683 ± 25	4392 ± 116	800 ± 100	36.3 ± 3.6
**9r** non-fused	10.1	—	77	—	112 ± 10	65 ± 11	—	7.3 ± 1.2
**3r** fused	5.2	—	1325	Long time	1410 ± 5	—	1400 ± 200	97.1 ± 3.1
**5r** fused	6.1	—	9836	Long time	5470 ± 36	3590 ± 109	8100 ± 3000	73.8 ± 2.4
**9r** fused	8.4	—	1427	Long time	1300 ± 11	—	—	40.4 ± 1.3

**Fig. 5:**
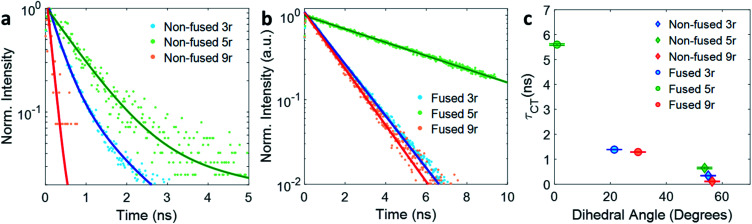
Exponential fits of the CT decay for (a) non-fused and (b) fused dimers. (c) Lifetime *τ* of the CT state plotted *versus* thienoacene–PDI dihedral angle.

The kinetic traces at the charge transfer wavelengths for the non-fused and fused series are plotted in Fig. 5a and b, respectively. In general, comparing across the two figures, the fused dimers each have a longer lifetime than their non-fused counterparts, as noted above regarding the **3r** dimers. From these figures we can also start to evaluate the effect of the linker length on the CT lifetime. In both the non-fused and fused series, the **5r** dimer has a substantially longer lifetime than either the **3r** or the **9r** dimers, breaking a potential trend with linker length. A possible explanation can be found by plotting the lifetimes *versus* the molecular dihedral angle, shown in Fig. 5c. The dihedral angle, calculated by DFT as the angle between the plane of the thienoacene linker and the plane of one of the PDIs, is dependent on the molecular structure. In the **3r** and **9r** dimers the ethylhexane side chain on the linker sterically interacts with the PDI, producing a severely twisted structure. However in the **5r** dimers there is one extra thiophene group separating the ethylhexane from the PDI, reducing the steric interaction and allowing the molecules to be more planar. This steric effect is moderate in the case of the non-fused dimers, and prominent in the case of the fused dimers, where ring fusion also enforces greater planarity. For the fused **5r** dimer, the dihedral angle is close to zero, and there is a striking increase in CT lifetime as compared to the other dimers. Interestingly, the fused **5r** dimer also has the highest efficiency in OPV devices out of the set of six,^15^ suggesting a possible link between excited state longevity and device performance.

With respect to linker length, although the alteration in the **5r** structure incumbers a direct comparison across the full length series, a comparison can still be made between the **3r** and **9r** dimers, which have similar steric hindrance but very different linker lengths. In the fused series, the **3r** and **9r** dimers have nearly the same lifetimes, suggesting that the CT lifetime is not particularly affected by the linker length on these length scales. Rather, the dihedral angle is a far more influential factor.

### Femtosecond transient absorption and DFT simulated excited state absorption spectra

**Fig. 6:**
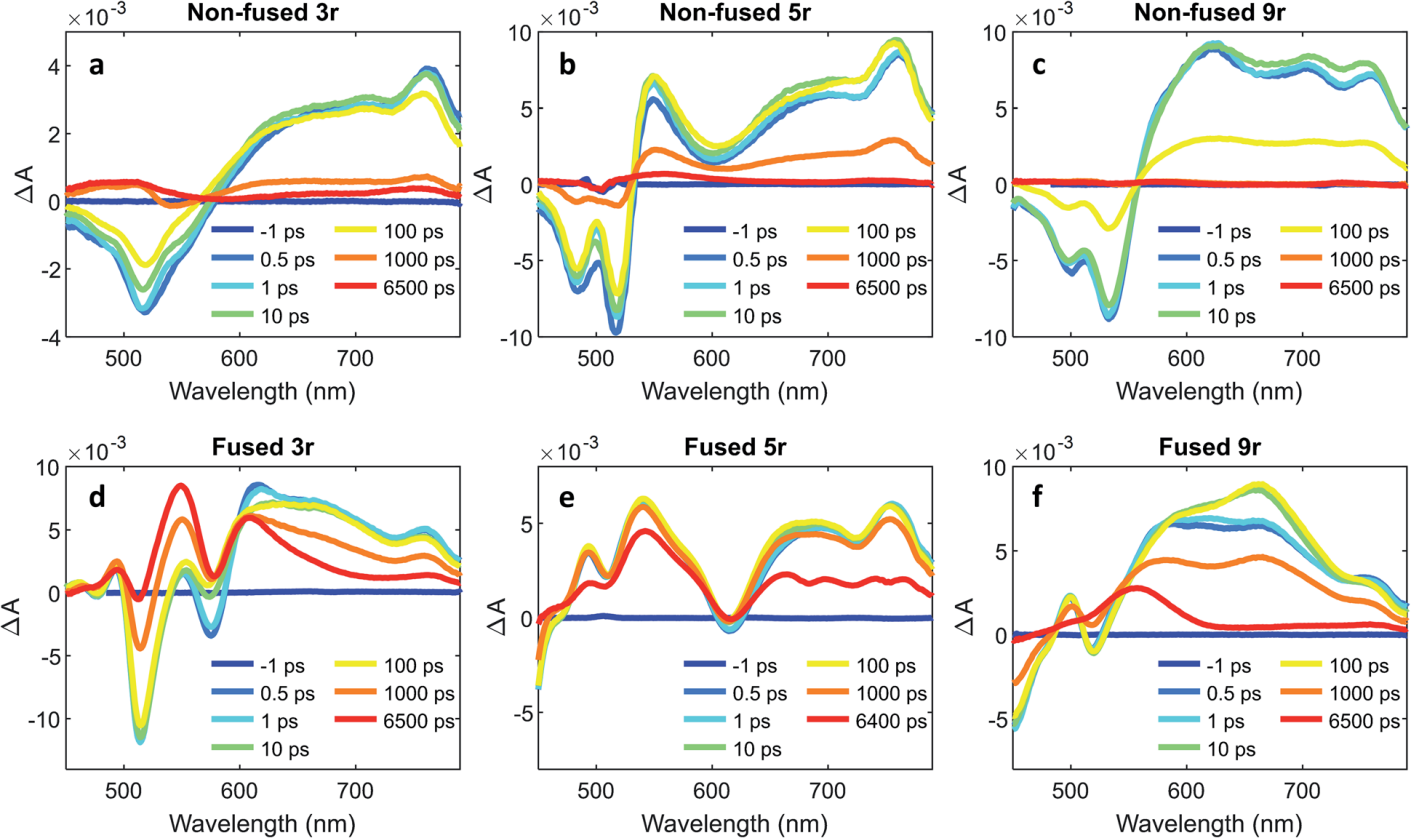
Wavelength traces from femtosecond transient absorption measurements with 500 nm pump at various timepoints for (a) non-fused **3r**, (b) non-fused **5r**, (c) non-fused **9r**, (d) fused **3r**, (e) fused **5r**, (f) fused **9r** dimers.

To further characterize the excited state dynamics on a sub-picosecond time scale, femtosecond transient absorption (fsTA) measurements were performed on the dimers. A standard pump-probe setup was used, with a 500 nm pump for selective excitation of the PDI unit and a white light probe. The wavelength traces of the resultant spectra at various probe time delays are shown in Fig. 6.

The non-fused spectra (Fig. 6a–c) are characterized by negative ground state bleach (GSB) features in the 450–575 nm region and positive excited state absorption (ESA) features above 575 nm. The ESA features grow in quickly within the first picosecond followed by a small amount of additional growth within the following ∼10 ps. The GSB and the ESA decay concurrently, with a small positive signal remaining at long time (6400 ps) that does not decay within the time frame of the experiment.

In the fused spectra the GSB features are again visible at early time between 450 and 600 nm, although in the case of the **5r** and **9r** dimers they are overwhelmed by the positive ESA features. As with the non-fused dimers, the initial GSB and ESA occur within 1 ps. There is again a fast process within the next 10 picoseconds, but it manifests differently in the spectrum for each dimer: in the fused **3r** the CT bleach at 575 nm and the sharp feature at 615 nm diminish; in the fused **5r** there is a slight GSB recovery between 600 and 700 nm; and in the fused **9r** there is a peak that grows in at 660 nm. In all three spectra there is a long-lived induced absorption centered around ∼550 nm which appears structured due to superposition with the GSB features. Additional nanosecond TA measurements show this feature decaying over tens of microseconds (ESI Fig. 4 and 5†) under deoxygenated conditions. Based on its exceptionally long lifetime and spectral location, we assign this absorption to the PDI triplet, which broadly absorbs in the 400 to 600 nm wavelength range.^49^,^55^

A global analysis was used to ascertain the lifetimes associated with the various process observed in the fsTA spectra. The decay associated species for each dimer are shown in ESI Fig. 6,† and the lifetime for each species is given in Table 1. For the non-fused dimers, because the overall shape of the spectrum is relatively unchanged during the first ∼100 ps (no significant new spectral features emerge), we suggest that the transition to the CT state occurs very fast, within the first picosecond, and the subsequent ∼10 ps process (*τ*_1_) is attributed to structural relaxation in the CT state. The dominant transition in the non-fused dimers is the relaxation of the ESA alongside the recovery of the GSB signal, which occurs over several hundred picoseconds (*τ*_3_). The lifetimes observed here are consistent with those observed for the CT emission band in the time-resolved emission data and lends further support to this transition being the recovery of the CT state. Although most of these excitons return directly to the ground state, there is a long-lived positive feature (*τ*_4_) in the 500 to 550 wavelength region in the **3r** and **5r** non-fused spectra which suggests that a small number of the excitons transition to the triplet state before returning to the ground state. There is also a small contribution from a longer-lived species in the 650–750 nm range (folded into *τ*_4_) which could be the PDI singlet emission. The fact that this is only a minor contribution suggests that most of the non-fused dimers follow the CT route, and the relatively strong PDI singlet emission observed in the steady state and time-resolved emission is a result of the singlet's comparatively high quantum yield.

**Fig. 7:**
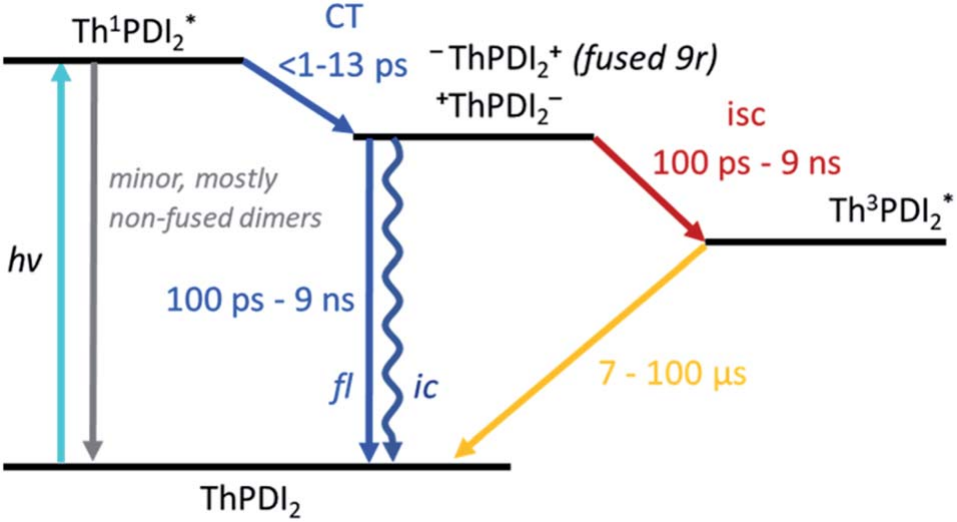
Energy diagram depicting the evolution of the excited state of the PDI dimers.

Turning to the fused dimers, the fused **3r** and **9r** dimers appear to transition more slowly to the CT state based on the aforementioned changes in the spectral features occurring in the first ∼10 ps (*τ*_1_). The fused **5r** dimer appears to undergo CT more rapidly (<1 ps) akin to the non-fused dimers. As compared to their non-fused counterparts, the fused dimers have a much longer ESA relaxation lifetime, occurring on the order of nanoseconds (*τ*_3_) as was also observed in the streak camera emission lifetimes. Based on a qualitative comparison of the relative peak intensities of the ESA and triplet absorptions, a much larger fraction of the CT excitons in the fused dimers undergo intersystem crossing (*τ*_4_) than in the non-fused dimers. An energy diagram showing the proposed excited state progression is shown in Fig. 7.

Conspicuously absent in many of the spectra is evidence of the formation of a PDI anion (∼710 nm absorption), which we might expect to see given the charge transfer from linker to PDI. The absence of this feature is perhaps a testament to the delocalization of both the HOMO and the LUMO as predicted in the MO diagrams (Fig. 2, ESI Fig. 2 and 3†). Although the MO diagrams do show a shift in electron density from the linker to the PDI units (from the HOMO to the LUMO), considerable electron density remains over the linker in the LUMO, particularly for the **3r** and **5r** dimers. In the non-fused **9r** spectra, there is a small peak at ∼710 nm buried in the broad and congested ESA, which could indicate stronger PDI anion formation and greater localization of electron density on the PDI unit when a longer linker is present. It is possible that a stronger effect would be observed with an even longer linker. In the fused **9r** spectrum the ESA growth observed at ∼650 nm is also distinctive. The relatively slow onset of this feature (8.4 ps) as compared to the inferred sub-picosecond CT occurring in the non-fused **9r** could be indicative of the previously discussed CT direction reversal.

**Fig. 8:**
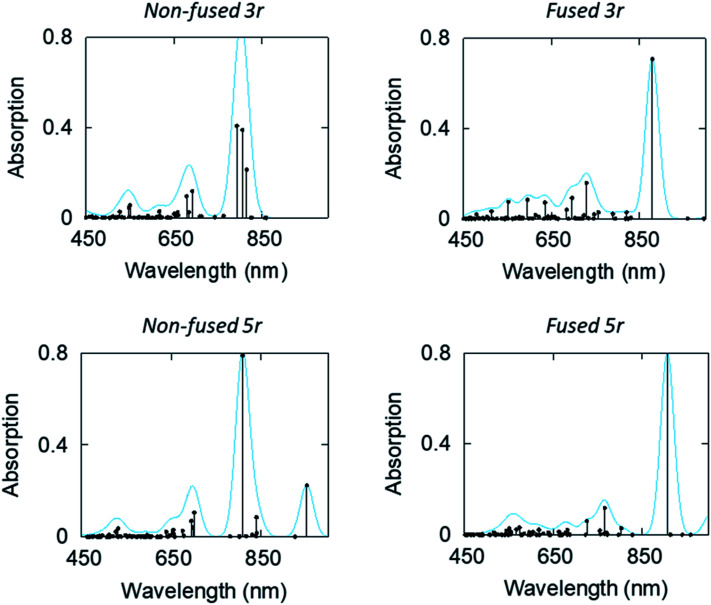
Computed ESA (in terms of oscillator strength, atomic units) of the first excited states of the non-fused and fused, **3r** and **5r** dimers.

Linear-response time-dependent DFT calculations were also performed to simulate the excited state absorption spectra of the 3- and 5-ring PDI dimers. These calculations reconstruct the observations in the optical transient absorption spectroscopic measurements and provide added insight into the electronic origins of these transitions. Fig. 8 shows the estimated excited-state absorption spectra of the first excited singlet of the **3r** and **5r** molecules. These calculations were performed using second linear response TDDFT;^56^,^57^ details of the calculations are presented in the ESI.† Selected spectra of other excited states are shown in ESI Fig. 7–10.†

We note, in agreement with the experimental observations, the presence of multiple absorption bands in the range 550–800 nm. This indicates multiple excited states contribute to the transient absorption. However, the ESA spectra of the CT states show that these have a strong absorption band in the 850–950 nm range, which is noticeably higher than the bands of other states. IR TA measurements (ESI Fig. 11 and 12†) confirm the existence of such a band for each of the dimers, which highlights the possibility of a distinctive peak separate from the PDI anion that may be used to identify the CT states.

## Summary and conclusions

In this study we have investigated the effects that ring fusion, linker length, and twist angle have on the electronic structure and the excited state evolution of a series of A–D–A PDI–thienoacene dimers. There is a clear energetic trend with linker length in both the non-fused and fused series, whereby the CT transition becomes progressively lower in energy as the linker length increases. Our TDDFT calculations showed the possibility of a reversal of charge transfer in the fused **9r** system, from PDI units into the linker, likely due to mixing of the energetically similar PDI and thienoacene MOs. Such reversals demonstrate how structural changes can dramatically alter the energetics of these organic molecules in ways that may not be immediately obvious. The implications of this for donor–acceptor charge separation efficiency and dynamics within bulk heterojunction films could be of interest for future study. Counterintuitively, there is little observed trend in CT lifetime with the linker length, although there is some hint that length starts to become a factor with the **9r** dimers, as we observe an additional peak in the fsTA data that is not present in the other dimers. It is possible that a linker length dependence would become apparent over even longer thienoacene chains.

Ring fusion and twist angle, on the other hand, have been shown to greatly influence the excited dynamics. Incorporation of the ring fusion element leads to more uniform population behavior (as compared to non-fused counterparts), effectively funneling energy from the higher lying transitions to the lowest energy CT state. Ring fusion also greatly stabilizes the CT state, allowing electron delocalization across the bridge–PDI interface, and increasing the relaxation lifetime by as much as a factor of ten. The dihedral angle is also an influential factor on the CT longevity, with the relative planarity of the fused **5r** dimer affording better MO overlap and a 5× longer lifetime than the corresponding **3r** and **9r** dimers. It is notable that the **5r** dimer has both the longest lifetime and the highest device efficiency, hinting at a connection between the molecular structure and the in-device performance. In addition, the calculated excited-state absorption spectrum of the charge transfer state shows a distinctive peak in the 800–900 nm region, which could be further investigated for the detection of this state.

In summary, the results have determined that many of the structural modifications to PDI acceptors that have previously been identified as favorable for device-driven metrics such as open circuit voltage and film morphology, also have significant implications for intrinsic photoinduced molecular behavior. Knowledge of these photophysical effects could be leveraged to further optimize these organic electron acceptors.

## Methods

### Sample preparation

The PDI dimers were synthesized according to the methodology described by Cai, *et. al*.^51^

### Steady state measurements

Steady-state UV-visible absorption spectra were taken with a Shimadzu UV-3600 UV-vis NIR spectrophotometer. Samples were diluted in chlorobenzene and measured in 2 mm quartz cuvettes. Steady-state fluorescence spectra were taken with a Horiba Scientific Fluorolog-3 spectrometer. Samples were diluted in chlorobenzene and measured in 1 cm glass cuvettes.

### Streak camera and femtosecond transient absorption measurements

Ultrafast time-resolved photoluminescence and transient absorption measurements were performed using a 35 fs amplified titanium:sapphire laser operating at a 2 kHz repetition rate. In both cases the sample was photo-excited with 500 nm photons generated from an optical parametric amplifier. For the ultrafast photoluminescence measurements, emitted photons were directed to a 150 mm spectrograph and streak camera. For the TA measurements, a white light probe was generated from 800 nm pulses focused on a sapphire plate. A mechanical delay stage was used to offset the pump pulse in time relative to the probe. Samples were diluted in chlorobenzene to an OD of ∼0.3 at 500 nm in 2 mm quartz cuvettes.

### Nanosecond transient absorption measurements

Measurements performed using an amplified titanium:sapphire laser (Spectra Physics Spitfire). The sample was photo-excited with 500 nm photons generated from an optical parametric amplifier. The probe was generated using a continuum light source (EOS from Ultrafast Systems) and transmitted light was collected *via* a spectrograph with visible (Si) array detector. The repetition rate of the system was 1 kHz with time resolution of 100 ps. Samples were diluted in chlorobenzene to an OD of ∼0.6 at 500 nm in 2 mm quartz cuvettes and were deoxygenated using the freeze–pump–thaw method prior to testing.

### Computational methods

We performed linear-response TDDFT calculations with the computational suite NWChem.^58^ We considered initially the exchange-correlation functionals B3LYP, LC-PBE, and CAM-B3LYP, and tested these functionals against the experimental, optical absorption data of the non-fused and fused **3r** systems. We found that the B3LYP functional performs well in the whole experimental range, except for the CT excitation energy which is underestimated for the non-fused systems. To compute the absorption spectra we employed the basis set 6-31G. A test case with 6-31G* shows these two basis sets perform similarly, although 6-31G is less computationally demanding, especially for the **5r** and **9r** systems, and convenient as these calculations require between 100 and 170 roots to cover the experimental absorption range. We optimize the structures initially using the ADF program, with the PBE XC functional and basis set DZP, frozen approximation employed too. The SCF energy convergence threshold was set as 10^–7^, with a fine integration grid. For the TDDFT calculations in NWChem we use an SCF convergence threshold of 10^–6^, and a Davidson threshold 10^–4^. These calculations were also carried out within the Tamm–Dancoff approximation. To estimate the absorption spectra of the excited states we employed our in-house “second linear response” module to compute the oscillator strengths corresponding to transitions between different excited states.^56^,^57^ Our calculations use the method based on auxiliary wave functions, as discussed by Tavernelli *et al.*^59^

### Data analysis

The kinetic fits for the streak camera data were performed using the trust region exponential fitting algorithm in MATLAB. For the non-fused **3r** and **5r** and fused **3r** dimers, the full spectrum was averaged to improve the signal to noise ratio, and then a single or biexponential fit was applied. For the remaining samples, the data around the wavelength of interest were averaged together (non-fused **9r** PDI: 550–600 nm, non-fused **9r** CT: 837–872 nm; fused **5r** PDI: 525–575 nm, fused **5r** CT: 650–750 nm; fused **9r** CT: 650–750 nm). Again, a single or biexponential fit was applied. A similar method was used for the nsTA data, averaged around the wavelength of interest indicated in ESI Fig. 4 and 5.†

The fsTA data was first corrected for background and chirp, and was then analyzed using a global analysis with a kinetic model of the following form:
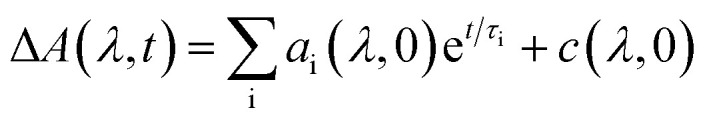
In this model, the transient absorption Δ*A* at any wavelength and time can be expressed as the sum of i basis sets *a*, each exponentially decaying with a lifetime *τ*, plus a constant *c*. Initial guesses for the number of basis sets i and the lifetimes *τ* were input into the model, and then the values of *a* and *τ* were varied iteratively to minimize the residuals between the model and the data.^60^ The lifetimes *τ* were fit globally, while the preexponential factors *a* were allowed to vary with wavelength. To avoid any contribution from the instrument response (pump pulse width of ∼35 fs), only the data from one picosecond onwards was fit using this model.

## Author contributions

All authors contributed to this work and have given approval to the final version of the manuscript.

## Conflicts of interest

There are no conflicts to declare.

## Supplementary Material

Supplementary information
