# Supplementary material for: Photophysical implications of ring fusion, linker length, and twisting angle in a series of perylenediimide–thienoacene dimers
Source: Chem Sci. 2020 Jul 1;11(27):7133–43. doi: 10.1039/d0sc02862b (PMC7654190; doi:10.1039/d0sc02862b)
Supplement: Supplementary file 1 [file SC-011-D0SC02862B-s001.pdf]

Electronic Supplementary Material (ESI) for Chemical Science.
This journal is © The Royal Society of Chemistry 2020

Supplementary Information

Photophysical Implications of Ring Fusion, Linker Length, and Twisting Angle in a Series of Perylenediimide-Thienoacene Dimers

Ariel A. Leonard,[a] Martín A. Mosquera,[a] Leighton O. Jones,[a] Zhengxu Cai,[b] Thomas J. Fauvell,[a] Matthew S. Kirschner,[a] David J. Gosztola[d], George C. Schatz,[a]* Richard D. Schaller,[a,d]* Luping Yu,[c]* and Lin X. Chen[a,e]*

[a]*Department of Chemistry, Northwestern University, 2145 Sheridan Road, Evanston, Illinois 60208, USA*
[b]*Beijing Key Laboratory of Construction Tailorable Advanced Functional Materials and Green Applications, School of Materials Science & Engineering, Beijing Institute of Technology, Beijing 100081, China*
[c]*Department of Chemistry and James Frank Institute, The University of Chicago, 929 East 57th Street, Chicago, Illinois 60637, USA*
[d]*Center for Nanoscale Materials, Argonne National Laboratory, 9700 South Cass Avenue, Lemont, Illinois 60439, USA*
[e]*Chemical Sciences and Engineering Division, Argonne National Laboratory, 9700 South Cass Avenue, Lemont, Illinois 60439, USA*

1. Solvatochromism of PDI Dimers

In order to aid in the assignment of the charge transfer peak, absorption spectra were collected on the dimers in chlorobenzene and chloroform; the energy of a charge transfer absorption band is known to be affected by the polarity of the solvent.[1] The spectra are plotted in SI Figure 1, and a bathochromic shift is observed in chloroform, which has the higher polarity. This is consistent with the higher polarity solvent stabilizing the charge transfer excited state, causing the shift to lower energy.

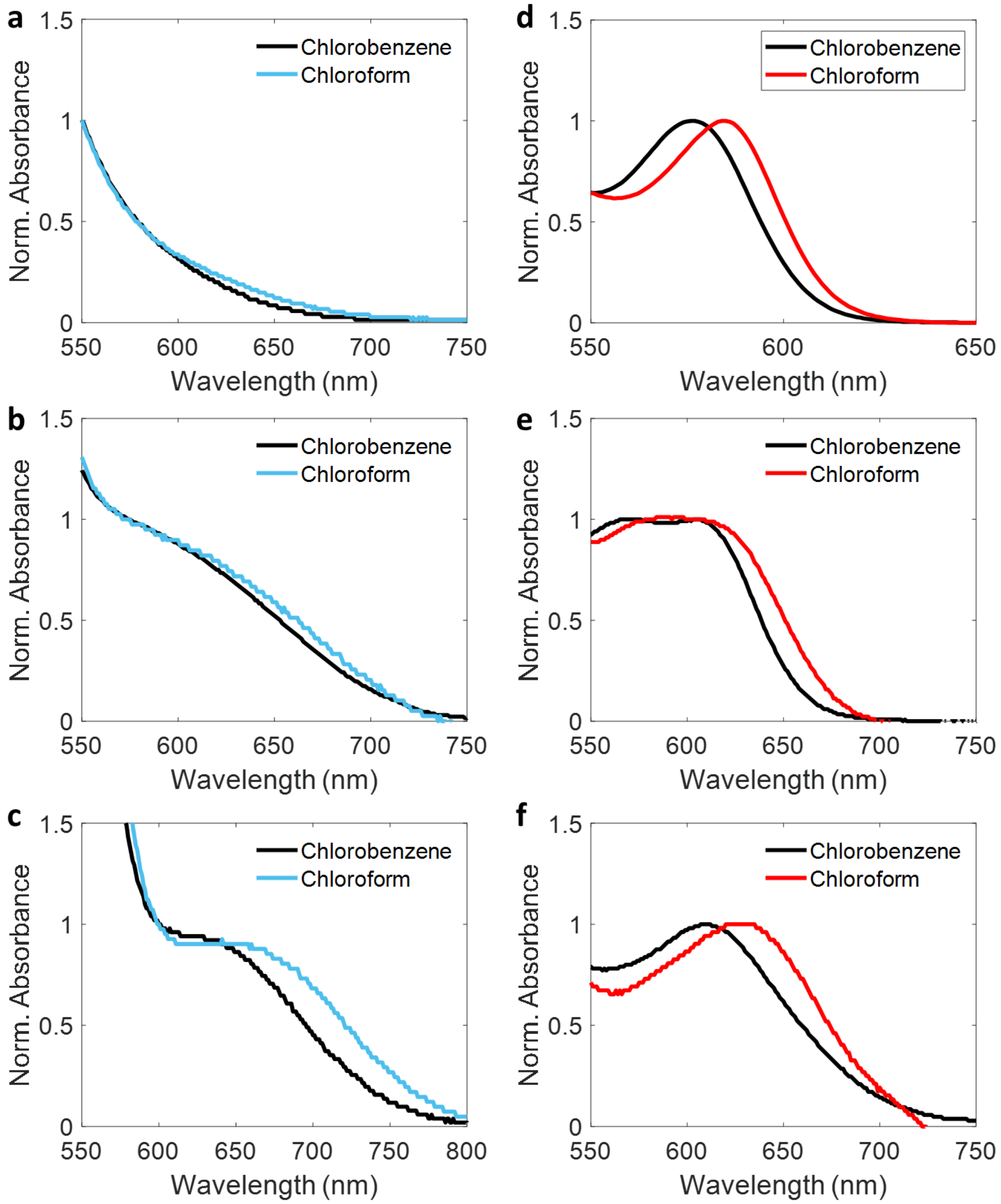

SI Figure 1. Absorption spectra of a) non-fused 3r, b) non-fused 5r, c) non-fused 9r, d) fused 3r, e) fused 5r, and f) fused 9r taken in chlorobenzene and chloroform. A bathochromic shift of the CT peak is observed in chloroform. Spectra were normalized at or near the CT peak.

2. Assignments of Ground State Transitions from DFT Calculations

Non-fused 3r

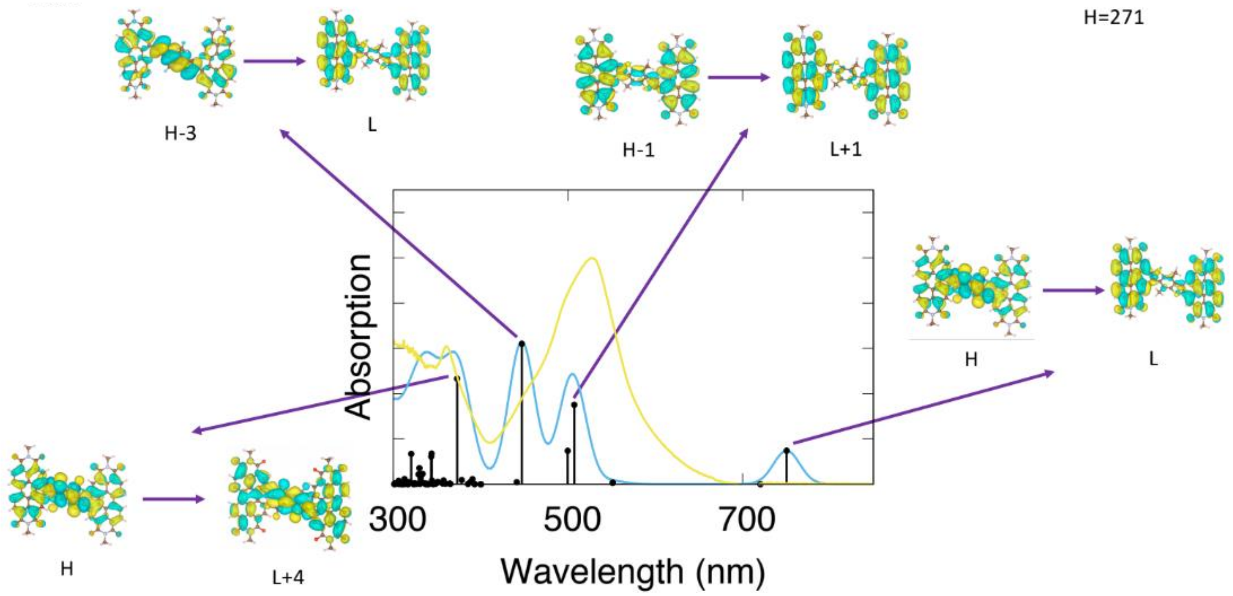

Fused 3r

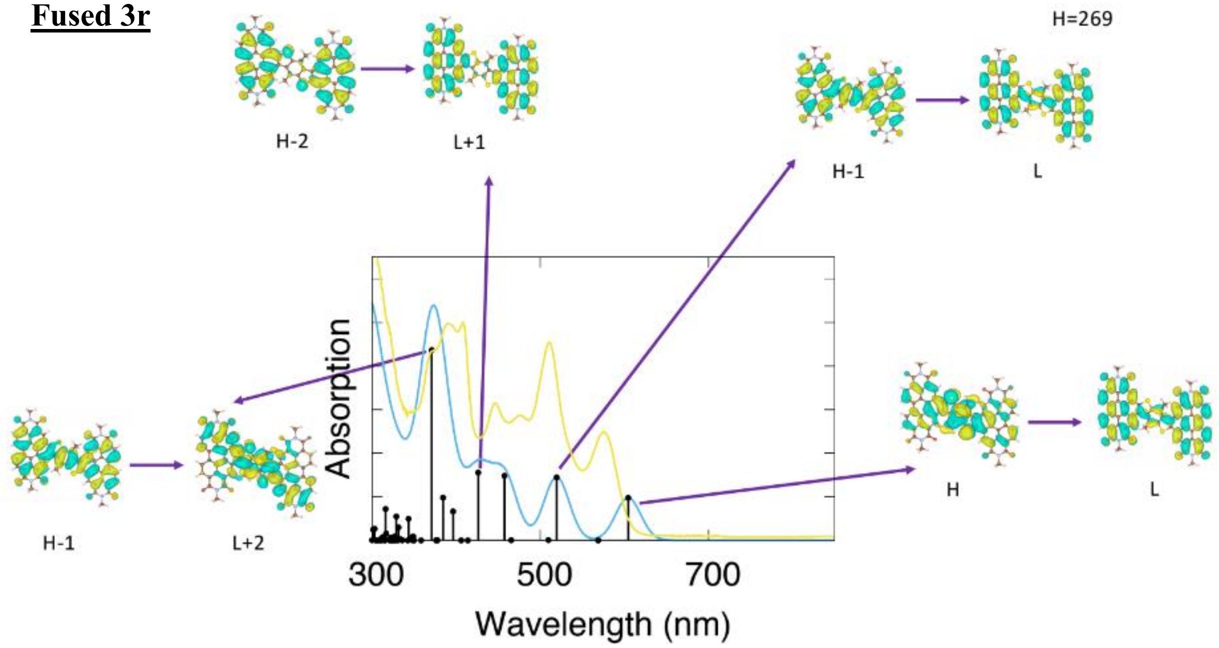

SI Figure 2. DFT calculated ground state absorption spectra (blue line) and molecular orbital transition assignments for non-fused 3r (top) and fused 3r (bottom) dimers. The experimental absorption spectra are shown (yellow line) for comparison.

Non-fused 5r

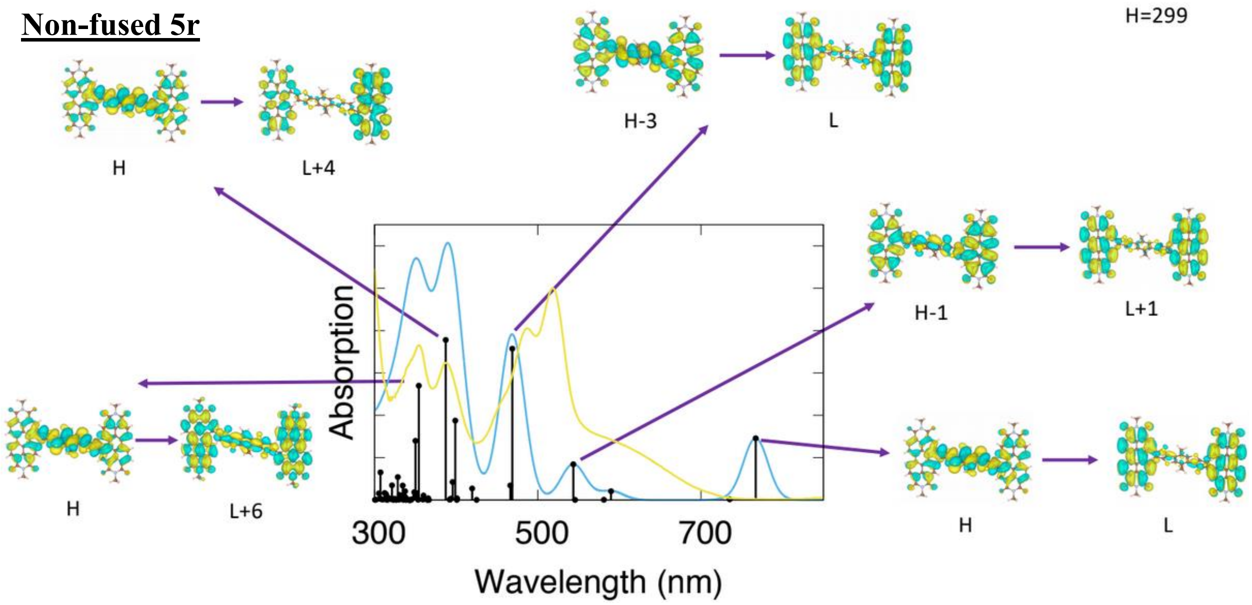

Fused 5r

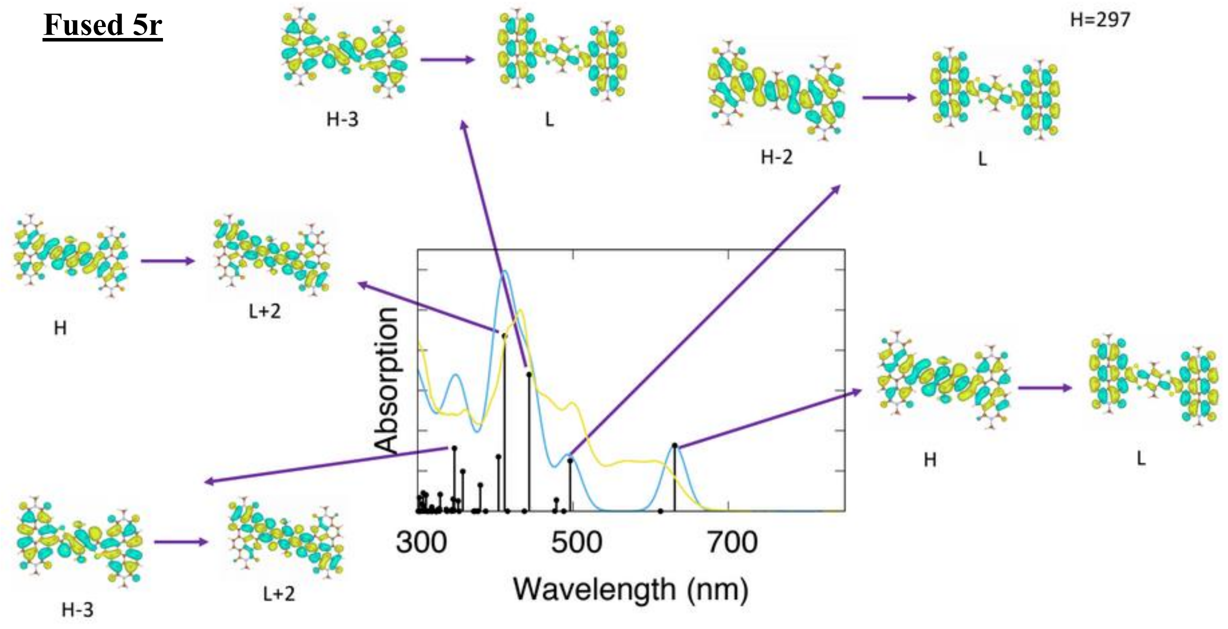

SI Figure 3. DFT calculated ground state absorption spectra (blue line) and molecular orbital transition assignments for non-fused 5r (top) and fused 5r (bottom) dimers. The experimental absorption spectra are shown (yellow line) for comparison.

SI Table 1. Frontier orbital energy (HOMO-LUMO, $\Delta E_{\text{H-L}}$) gaps and optical gaps ($\Delta E_{\text{H-L}}$) computed with B3LYP/6-31G.

	Non-fused 3r	Non-fused 5r	Non-fused 9r	Fused 3r	Fused 5r	Fused 9r
$\Delta E_{\text{H-L}}$ (eV)	1.98	1.89	1.55	2.41	2.23	2.02
$\Delta E_{\text{H-L}}$ (nm)	626	656	800	515	556	614
ΔE_{opt} (eV)	1.65	1.62	1.31	2.05	1.97	1.78
ΔE_{opt} (nm)	752	765	947	605	629	697

3. Long-Time Characterization with Nanosecond Transient Absorption

In order to characterize the long-lived state observed in the femtosecond transient absorption measurements, nanosecond transient absorption (nsTA) was performed on the dimers. The results are plotted in SI Figures 4 and 5. The early time wavelength traces for the dimers contain the same features observed in the terminal fsTA wavelength traces. The kinetics were fit to a single or biexponential model at the specified wavelengths, which were chosen based on the maximum of the excited state absorption feature with the exception of the non-fused 9r dimer. The lifetimes are reported in Table 1 of the manuscript. The microsecond-scale lifetimes are attributed to a long-lived triplet state. The triplet lifetime is longer in the fused dimers as compared to the non-fused dimers.

With regards to the non-fused 9r dimer, it appears that the ground state bleach for the non-fused 9r dimer at 441 nm weakly persists into the microsecond regime. This feature bleach feature has been fitted and the lifetime is included in Table 1 for completeness. A possibility is that there is some small amount of triplet formation in the non-fused 9r dimer population, as has been observed for the other dimers, but the excited state absorption is buried in the noise. If this is the case, then the ground state bleach at 441 nm would give an approximation of this triplet lifetime.

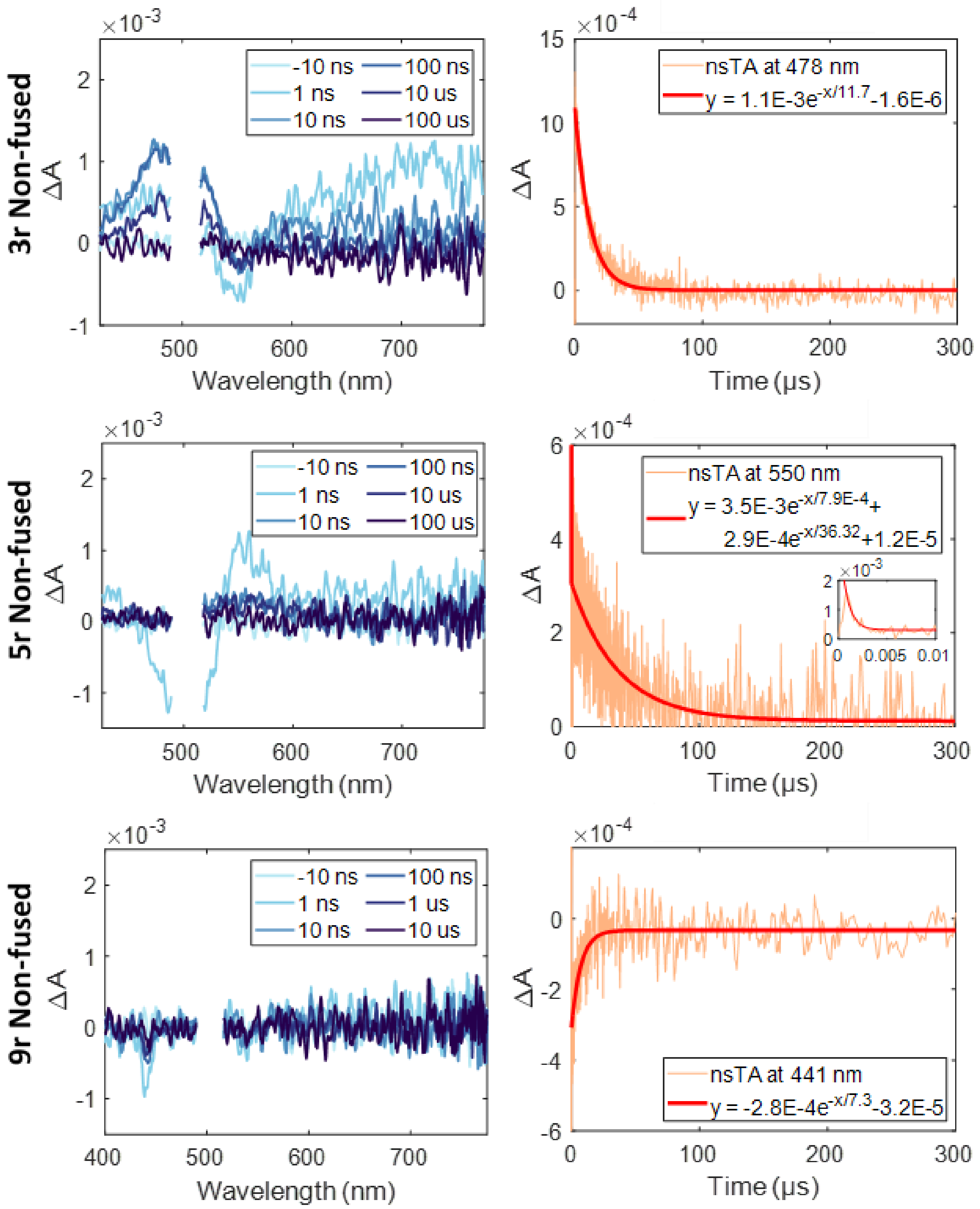

SI Figure 4. Nanosecond transient absorption spectra of 3r non-fused (top), 5r non-fused (middle), and 9r non-fused (bottom) dimers. Wavelength traces at various time points after excitation are plotted on the left and kinetic traces at the specified wavelengths are plotted on the right.

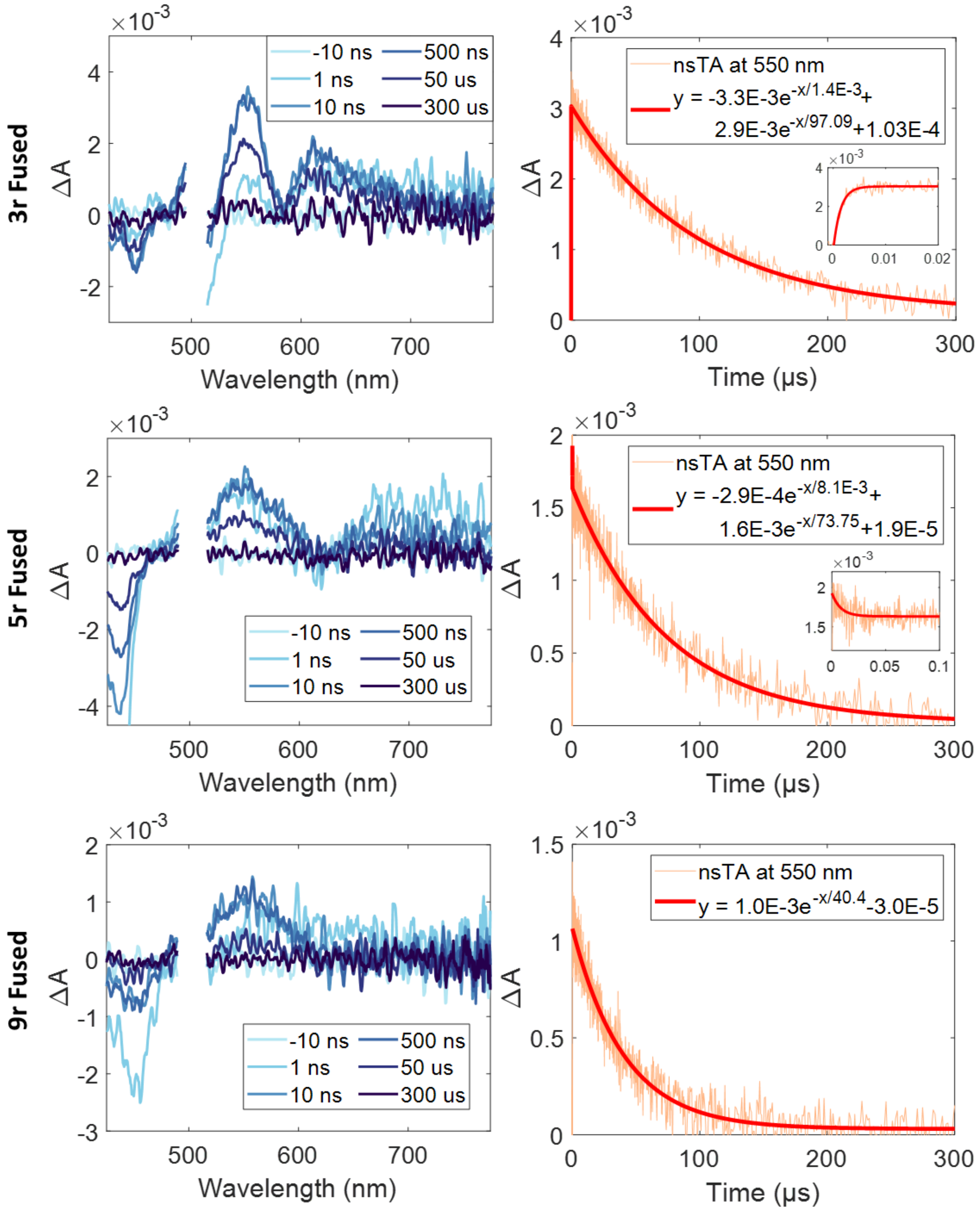

SI Figure 5. Nanosecond transient absorption spectra of 3r fused (top), 5r fused (middle), and 9r fused (bottom) dimers. Wavelength traces at various time points after excitation are plotted on the left and kinetic traces at the specified wavelengths are plotted on the right. In the wavelength traces, the portion of the ΔA spectrum around the 500 nm excitation pump is omitted for ease of viewing the overall line shape.

4. Global Analysis of Femtosecond Transient Absorption Spectra

The decay associated species from global fitting of the fsTA spectra are shown in SI Figure 6. The global fit was performed as described in the Methods section. The non-fused 3r and all of the fused dimers were fit to a model with $i = 3$ decay associated species, while the non-fused 5r was fit with $i = 4$ and the non-fused 9r was fit with $i = 2$. In all but the non-fused 9r dimer, a long-lived triplet species much longer than the time window of the experiment (6500 ps) was observed and is included as a static spectrum, however, the exponential decay is observed in the longer nsTA measurements (SI Figures 2 and 3). This species appears to be a very minor contributor for the non-fused 9r dimer and therefore is not included in the global fit for the molecule.

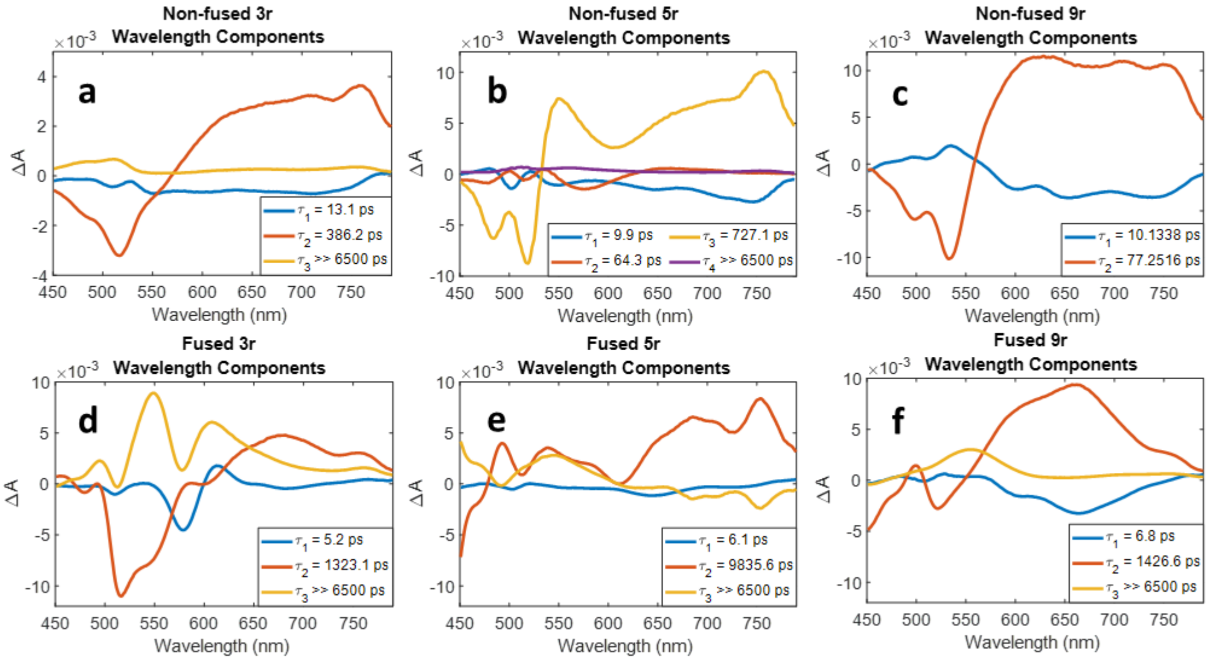

SI Figure 6. Decay associated species from global analysis of fsTA spectra on a) non-fused 3r, b) non-fused 5r, c) non-fused 9r, d) fused 3r, e) fused 5r, and f) fused 9r dimers.

5. Further Details on TDDFT Calculations

The excited-state absorption calculations, performed by determining amplitudes of transitions between excited-states, were carried out by employing the auxiliary wave functions that arise from the Casida formalism. We employ the Tamm-Dancoff approximation, which allows us to express linear-response eigenvalue problem as:

$$\boldsymbol{A}\boldsymbol{X}_I = \omega_I \boldsymbol{X}_I$$

Where $\boldsymbol{A}$ is the matrix with all the information about the orbital transitions: the energy differences between virtual and occupied orbitals, and their interactions by means of the Hartree-exchange-correlation first-order kernel. The variable ω_I denotes the excitation energy for a transition from the ground-state of the system to state *I*. With the excitation vector $\boldsymbol{X}_I$ with approximate the oscillator strength for a transition between excited states labeled *J* and *I* as follows:

$$f_{IJ} = \frac{2}{3}\left(\omega_J - \omega_I\right)\left|\langle \Phi_J | \hat{\boldsymbol{\mu}} | \Phi_I \rangle\right|^2$$

The auxiliary wave function for a given excited state J reads:

$$\Phi_J = \sum_{ai} X_{I,ai}\, \hat{a}^{\dagger}\hat{\imath}\, \Phi_0$$

Where the operation $\hat{a}^{\dagger}\hat{\imath}$ promotes an electron in the occupied spatial orbital i into the virtual level a. The oscillator strength is computed as described in our prior work, Eqs. (36)-(39).[2] Due to the size of the systems and the relatively high number of states to consider, we did not perform a second electronic relaxation,[2] but only performed computations with auxiliary wave functions.

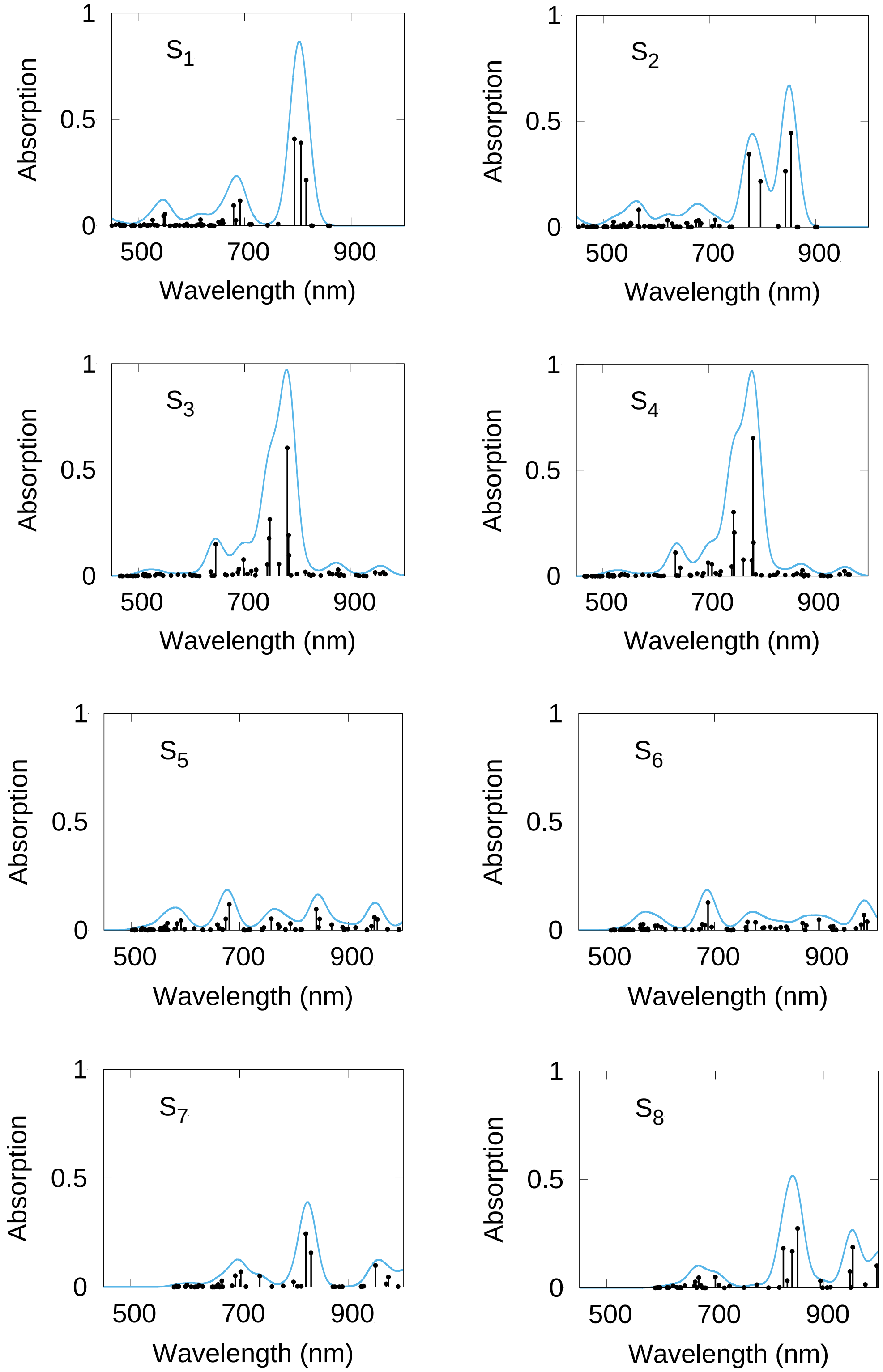

SI Figure 7. Computed absorption spectra of the first eight excited states of the 3-ring non-fused system, in the range 450-1000 nm. Absorption displayed in oscillator strength units.

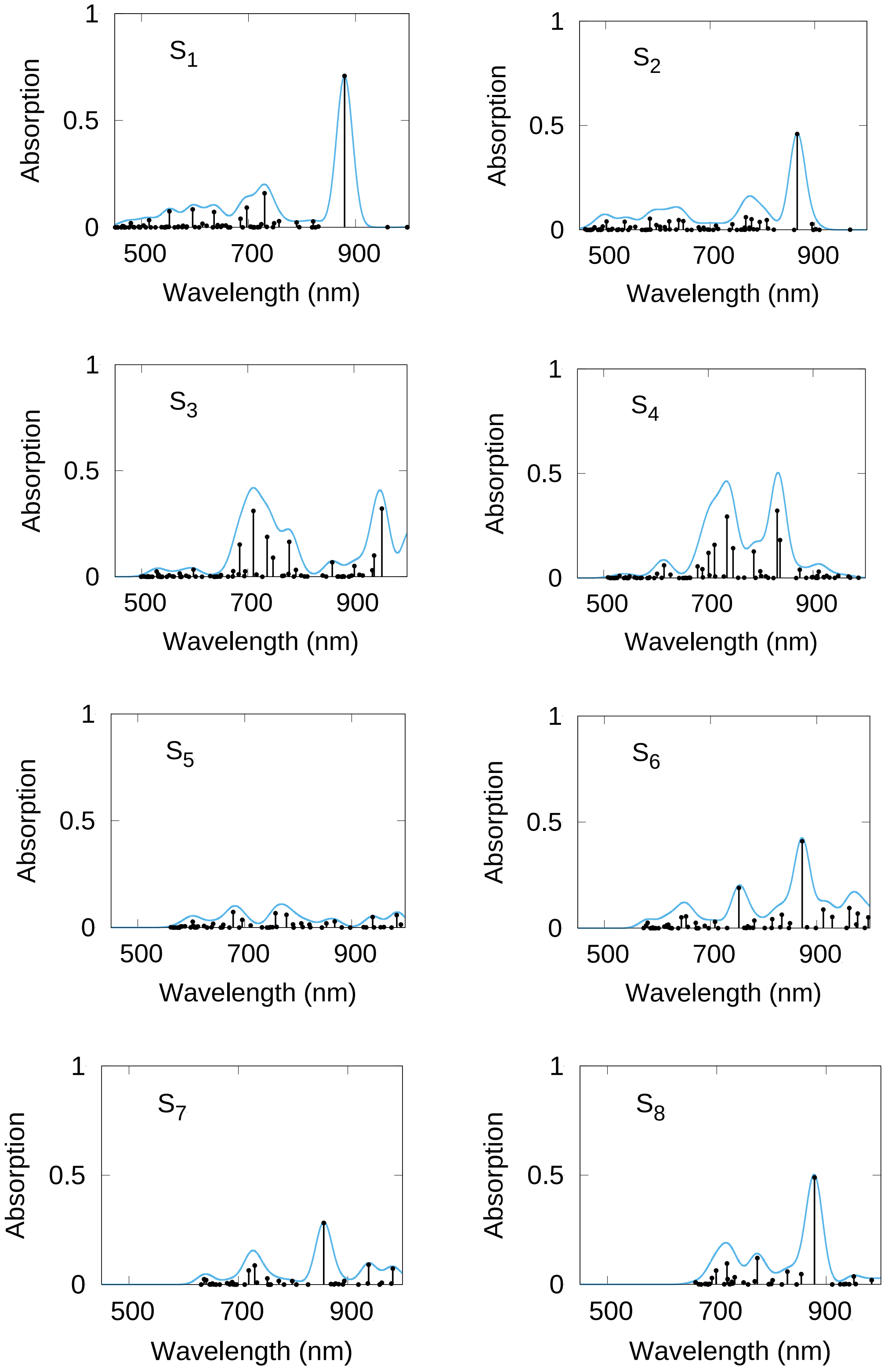

SI Figure 8. Computed absorption spectra of the first eight excited states of the 3-ring fused molecule, in the range 450-1000 nm. Absorption displayed in oscillator strength units.

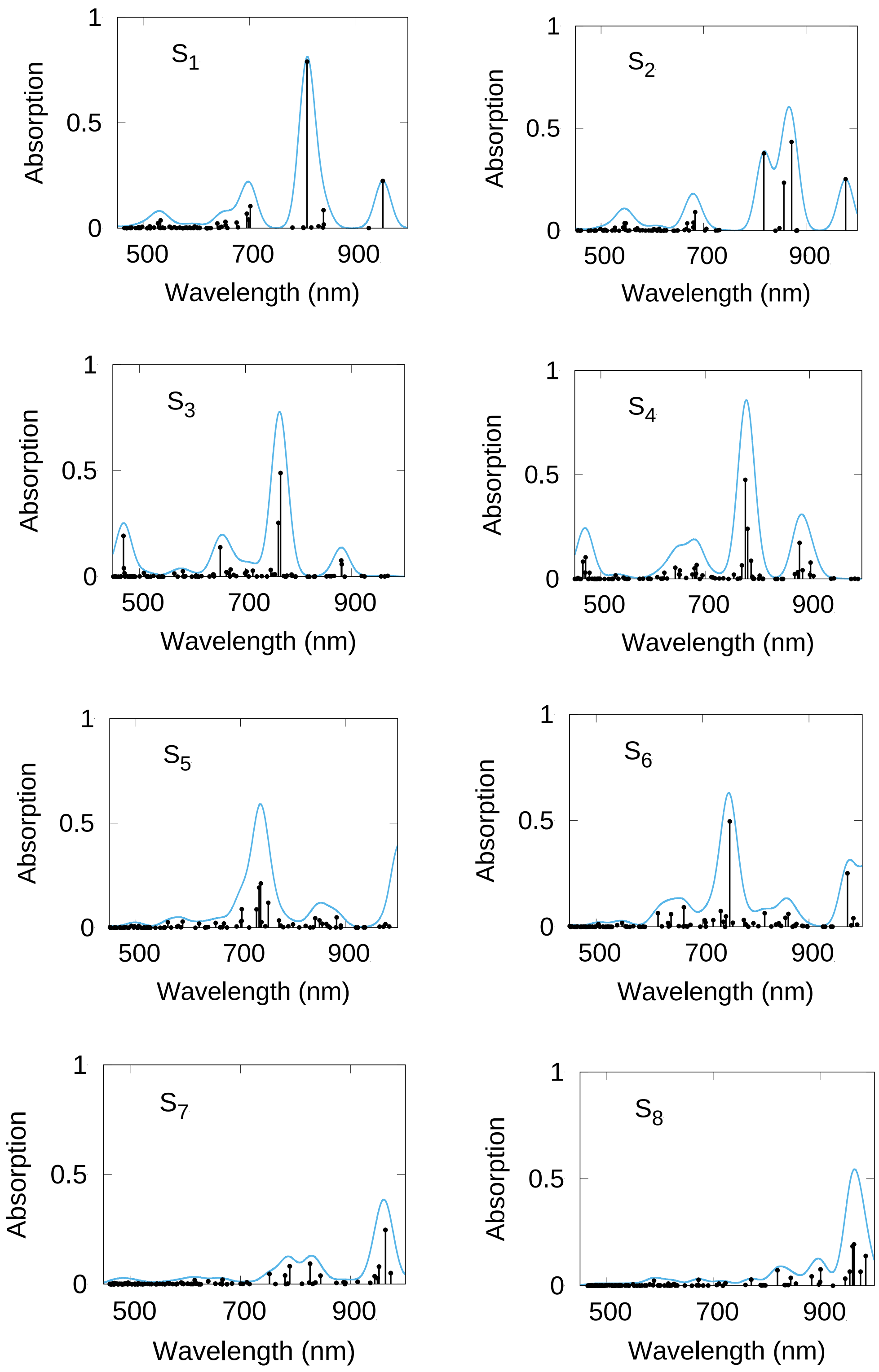

SI Figure 9. Computed absorption spectra of the first eight excited states of the 5-ring non-fused molecule, in the range 450-1000 nm. Absorption displayed in oscillator strength units.

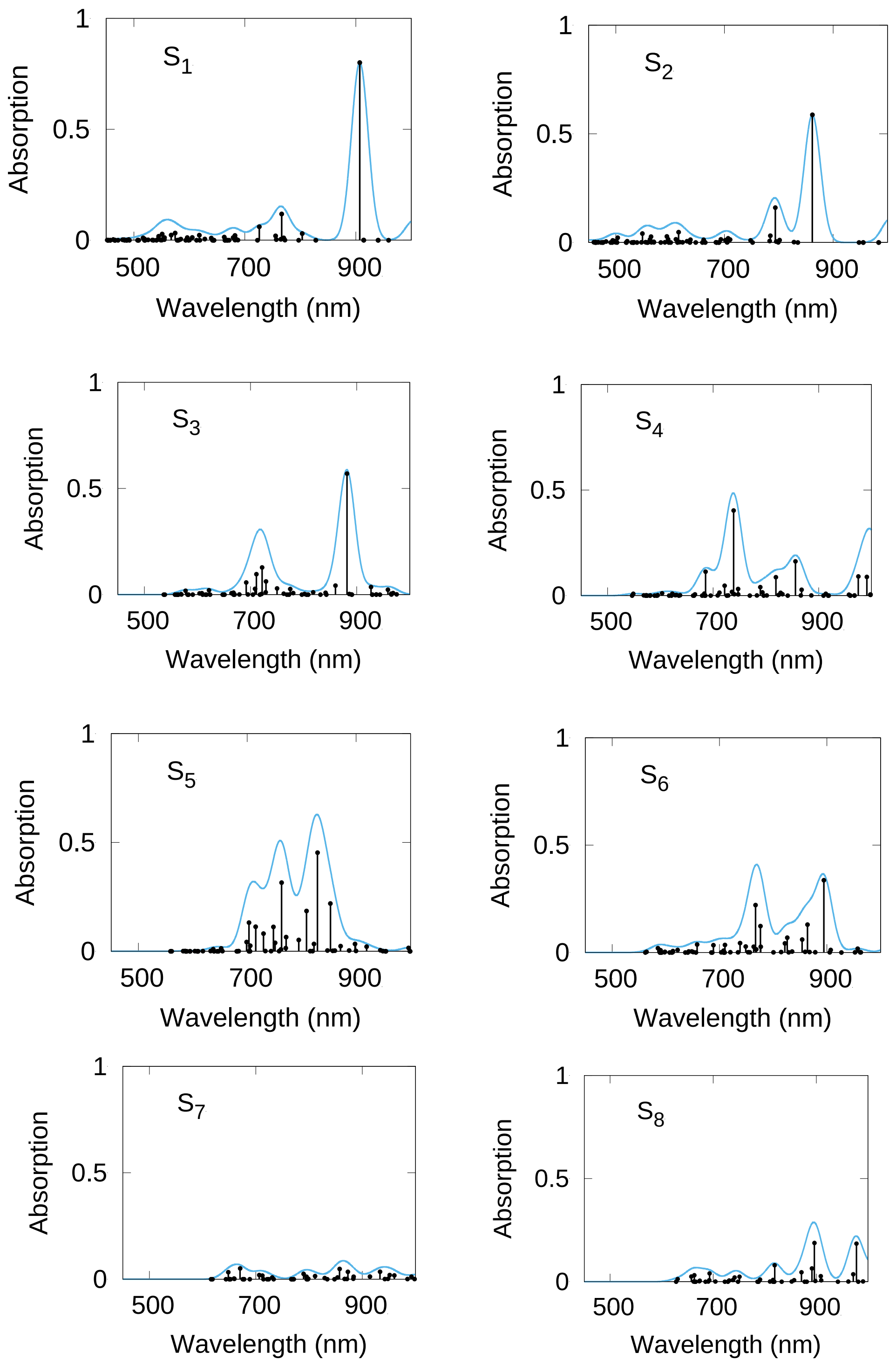

SI Figure 10. Computed absorption spectra of the first eight excited states of the 5-ring fused molecule, in the range 450-1000 nm. Absorption displayed in oscillator strength units.

5. IR Transient Absorption Spectroscopy

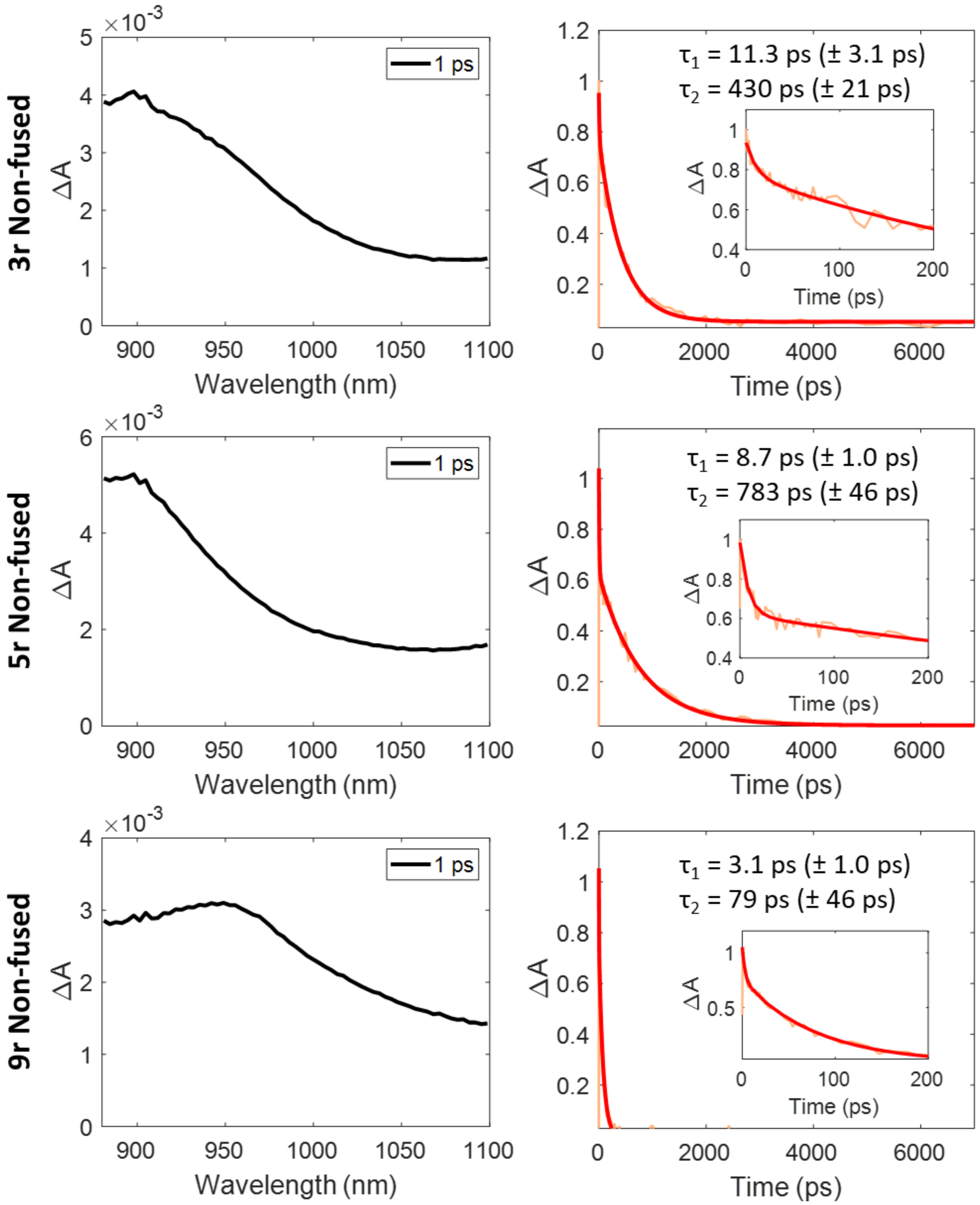

SI Figure 11. IR transient absorption spectra of 3r non-fused (top), 5r non-fused (middle), and 9r non-fused (bottom) dimers. Wavelength traces at 1 ps after excitation are plotted on the left and kinetic traces taken at the peak location in the region of 900 to 950 nm are shown on the right.

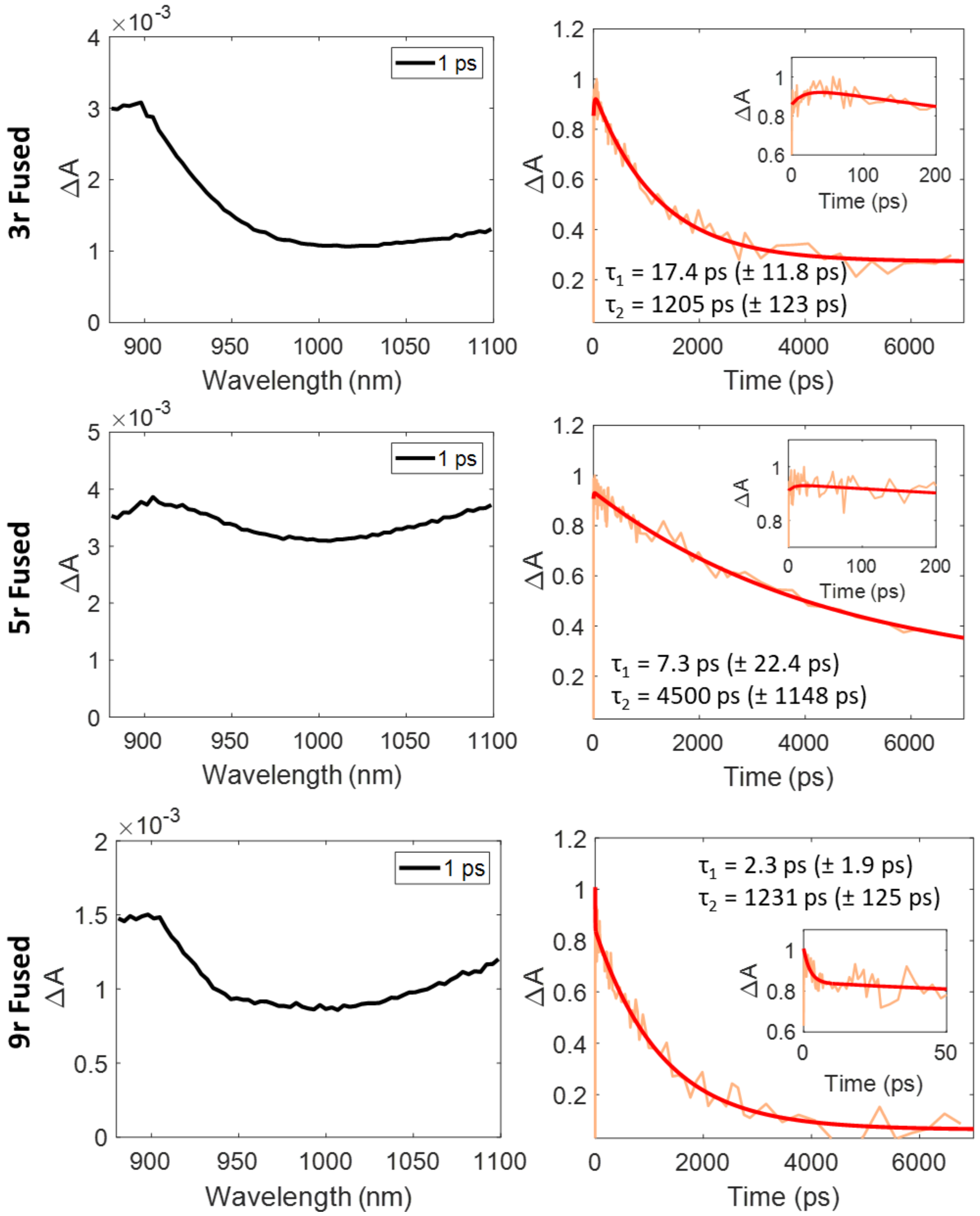

SI Figure 12. IR transient absorption spectra of 3r fused (top), 5r fused (middle), and 9r fused (bottom) dimers. Wavelength traces at 1 ps after excitation are plotted on the left and kinetic traces taken at the peak location in the region of 900 to 950 nm are shown on the right.

References

1 N. J. Turro, *Modern Molecular Photochemistry*, University Science Books, Sausalito, CA, 1991.

2 M. A. Mosquera, L. X. Chen, M. A. Ratner and G. C. Schatz, *J. Chem. Phys.*, 2016, **144**, 204105.
